# Genome-enabled predictions for binomial traits in sugar beet populations

**DOI:** 10.1186/1471-2156-15-87

**Published:** 2014-07-22

**Authors:** Filippo Biscarini, Piergiorgio Stevanato, Chiara Broccanello, Alessandra Stella, Massimo Saccomani

**Affiliations:** 1 Department of Bioinformatics, PTP, Via Einstein - Loc. Cascina Codazza, Lodi, Italy; 2 DAFNE, Università di Padova, 24105 Padova, Italy; 3 IBBA-CNR, Via Einstein, 26900 Lodi, Italy

**Keywords:** Genomic predictions, Binomial traits, Root vigor, Sugar beet

## Abstract

**Background:**

Genomic information can be used to predict not only continuous but also categorical (e.g. binomial) traits. Several traits of interest in human medicine and agriculture present a discrete distribution of phenotypes (e.g. disease status). Root vigor in sugar beet (*B. vulgaris*) is an example of binomial trait of agronomic importance. In this paper, a panel of 192 SNPs (single nucleotide polymorphisms) was used to genotype 124 sugar beet individual plants from 18 lines, and to classify them as showing “high” or “low” root vigor.

**Results:**

A threshold model was used to fit the relationship between binomial root vigor and SNP genotypes, through the matrix of genomic relationships between individuals in a genomic BLUP (G-BLUP) approach. From a 5-fold cross-validation scheme, 500 testing subsets were generated. The estimated average cross-validation error rate was 0.000731 (0.073%). Only 9 out of 12326 test observations (500 replicates for an average test set size of 24.65) were misclassified.

**Conclusions:**

The estimated prediction accuracy was quite high. Such accurate predictions may be related to the high estimated heritability for root vigor (0.783) and to the few genes with large effect underlying the trait. Despite the sparse SNP panel, there was sufficient within-scaffold LD where SNPs with large effect on root vigor were located to allow for genome-enabled predictions to work.

## Background

Most of current research and applications in genetics are driven by the large quantity of data on individual genomic polymorphisms produced by modern high-throughput genotyping and sequencing technologies [1]. A thriving area is that of genomic predictions in animal and plant science and human medicine.

Genomic data are used to predict future or unobserved events (e.g. disease risk [2]), or the unknown genetic component of given phenotypes (e.g. GEBVs -genomic breeding values- in livestock, crops and trees [3-5]). Such predictions are based on the entire available genomic information, irrespective of the position along the genome or point effects on the response. This ingenious and highly effective “black box” approach was conceived and first described by Meuwissen et al. around the turn of the millennium [6] and has since then found several applications and started fruitful areas of research.

Genomic information can in principle be used to predict continuous or categorical (ordered or unordered) polygenic traits. Most works so far focussed on continuous traits, while fewer studies dealt with genomic predictions for categorical traits [7-11]. However, several traits of interest in human medicine and agriculture present a discrete distribution of phenotypes (e.g. litter size in mammals), often binomial (e.g. disease status). Statistical methods used for genomic predictions of continuous traits cannot be adequately applied for such traits: the relationship between predictors and binomial phenotypes is logistic rather than linear; the phenotypes follow a binomial rather than normal distribution; the variance is no longer constant but a function of the expectation [12]. Root vigor in sugar beet (*B. vulgaris*) is an example of binomial trait of agronomic importance.

In this paper, SNP (single nucleotide polymorphisms) genotypes were used for the classification problem of predicting “high” or “low” root vigor inidividual plants in sugar beet.

Pioneering works on genomic predictions for continuous traits in sugar beet already exist [13,14]. However, this is the first study to propose direct modeling of genomic predictions for binomial traits in sugar beet and, to our knowledge, among the few to address this problem in plants in general.

## Methods

### Experimental population

A population of 124 individual sugar beet (*B. vulgaris*) plants from 18 high- and low-root-vigor lines were available. These lines were characterised by different productivity and were provided by Lion Seeds Ltd. (UK). Root vigor is related to nutrient uptake from the soil and plant productivity [15], and is recorded as a binary trait (either high or low). The lines were phenotyped by measuring the root elongation rate of eleven-days-old seedlings grown under hydroponic conditions. There was no predetermined root elongation rate threshold to classify a sugar beet as having high or low root vigour, and the decision was subjectively made upon phenotypic inspection. The classification has nevertheless been shown to be robust: seedlings classified as “low” or “high” maintain the same class also at the adult plant stage [15]. There were three low-root-vigor (24 individuals) and 15 high-root-vigor (100 individuals) lines. Root elongation rate was < 3 mm/day in the low-root-vigor lines and > 6 mm/day in the high-root-vigor lines.

### Marker genotypes and imputation

All individual plants were genotyped for 192 SNP markers with the high-throughput marker array QuantStudio 12K Flex system coupled with Taqman OpenArray technology. Additional details on the genotyping procedure are described in Stevanato et al., 2013 [16].

The initial genotype screening led to the detection of one duplicated individual (100*%* matching genotypes) from a high-root-vigor line, which was removed. The average per-sample and per-marker call-rate was 0.984 and 0.969. Only one SNP had a per-marker call-rate ≤ 85*%* and was removed from the analysis. There were in total 738 missing genotypes (3.14*%*). Missing genotypes were imputed based on linkage disequilibrium (LD, [17]). After imputation data were edited for minor allele frequency (MAF): 16 SNPs with MAF ≤ 2.5*%* were discarded. This left a total of 123 individuals and 175 SNP markers for the analysis. An overview of the data used in the paper is given in Table 1. Table 2 reports the distribution of the 175 SNPs (and related scaffolds) used in the analysis along the 9 chromosomes of the *Beta vulgaris* genome. The average scaffold size was 1037 kbps (range: 34.5 - 4957 kbps).

**Table 1:** Description of the experimental population and SNP marker genotypes

*N. plant samples*		124
	(duplicated)	1
*N. sugar beet lines*		18
	High-root-vigor lines	15
	Samples	100
	Low-root-vigor lines	3
	Samples	24
*N. SNPs*		192
*Average call-rate*		
	Per SNP	0.969
	Per sample	0.984
	N. of SNP call-rate ≤ 85*%*	1
*Average MAF*		0.262
	N. SNPs MAF ≤ 2.5*%*	16
	N. SNPs MAF ≥ 2.5*%*	175
		

**Table 2:** **Per-chromosome distribution of scaffolds and SNPs along the *Beta vulgaris * genome (“-” indicates scaffolds and SNPs not yet assigned to chromosomes)**

**Chromosome**	**# scaffolds**	**# SNPs**
1	6	8
2	7	11
3	10	18
4	18	33
5	9	16
6	9	16
7	14	21
8	7	10
9	10	22
-	9	20
Total	99	175

### Genomic relationships

Marker genotypes can be used for genome-enabled predictions either by directly estimating and summing their effects over all loci (Ridge Regression BLUP - RR-BLUP) or, indirectly, through the estimation of realized relationships between individuals (genomic BLUP - G-BLUP). These are two different parametrizations of the genomic selection model described in Meuwissen et al. [6]. The two approaches have been shown to be equivalent [18,19].

In this paper SNP genotypes were incorporated in the prediction model through the matrix of genomic relationships between individuals. From imputed genotypes the genomic relationships between individual plants were computed according to Van Raden (2008 [19]) as:

(1) $$G=\frac{ZZ^{\prime }}{2\sum p_{i}\left(1-p_{i}\right)}$$

where ***G*** is the matrix of genomic relationships, ***Z*** is the matrix of centered SNP genotypes per individual (-1, 0 or 1 for the homozygous, heterozygous and other homozygous respectively), and *p*_*i*_ is the allele frequency at SNP *i*. Genomic relationships were used to model covariances between observations and to evaluate the genetic structure of the population.

### Threshold model

In a GBLUP (Genomic Best Linear Unbiased Predictions) framework [6], the probability *Pr *(*Y *= [ 0/1]|*X*) of having either high or low root vigor given the predictors was modeled assuming a continuous underlying latent variable *l* (“liability”). A threshold model [12] of the following form was fitted:

(2) l=1μ+Xg+e

with ***l*** the vector of continuous gaussian liabilities, ***g*** the vector of additive genetic values of individual plants, and ***e*** the vector of logistically distributed residuals; ***X*** is a design matrix that allocates records to genetic values. The genetic and residual variances were $\text{Var}(g)=G\sigma_{a}^{2}$ and $\text{Var}(e)=I\frac{s^{2}\pi^{2}}{3}$ (variance of the logistic distribution, with scale parameter *s*=1; ***I*** is the identity matrix). The narrow sense heritability for root vigor was then estimated as:

(3) $$h^{2}=\frac{\sigma_{a}^{2}}{\sigma_{a}^{2}+\frac{\pi^{2}}{3}}=\frac{\sigma_{a}^{2}}{\sigma_{a}^{2}+3.29}$$

Low and high root vigor (coded as 0 and 1 respectively) phenotypes were the input of model 2, which returned a probability (for individuals with known or unknown phenotype) of belonging to either class. The probability of classifying each observation *i* into high- or low-root-vigor plant was obtained from the cumulative distribution function of the logistic distribution (i.e. the logistic function: $p_{i}=\text{logistic}(l_{i})=\frac{e^{\mu +g_{i}}}{1+e^{\mu +g_{i}}}$). Individuals were classified as high-/low-root vigour if *p*_*i *_>/≤ 0.5.

### Cross-validation

In order to obtain a valid estimate of the classification error from model (2), a 5-fold cross validation procedure was adopted [20]. The 123 samples were randomly split into 5 subsets of approximately the same size. In turn, the observations in one subset were set to missing and predicted using the model trained with the remaining four subsets, until all subsets were used once as validation set. This process was repeated 100 times, each time randomly sampling different subsets, eventually yielding 500 replicates of the analysis. The test error rate in each replicate was computed as:

(4) $${\text{ER}}_{\left(n\right)}=\frac{1}{n}\overset{n}{\underset{i=1}{\sum }}{\text{Err}}_{i}$$

where *n* is the number of observations in the test set and ${\text{Err}}_{i}=I\left(y_{i}\neq {ŷ}_{i}\right)$, with *I *(·) an indicator function which returns a value of 1 if the predicted and observed phenotypes are different, 0 otherwise. The cross-validation (CV) error rate was then estimated averaging the test error rate over all replicates:

(5) $${\text{CV}}_{k}=\frac{1}{k}\overset{k}{\underset{i=1}{\sum }}{\text{ER}}_{i}$$

### Software

The programme *Beagle* was used to impute missing genotypes [17]. The computer package for linear mixed models *Asreml* was used to fit the threshold model in (2) and estimate variance components [21]. Genomic relationships between plants were estimated with “ad hoc” *Python* code. Data preparation and figures were produced with the open source statistical environment *R*[22].

## Results

### Realized relationships

**Figure 1:**
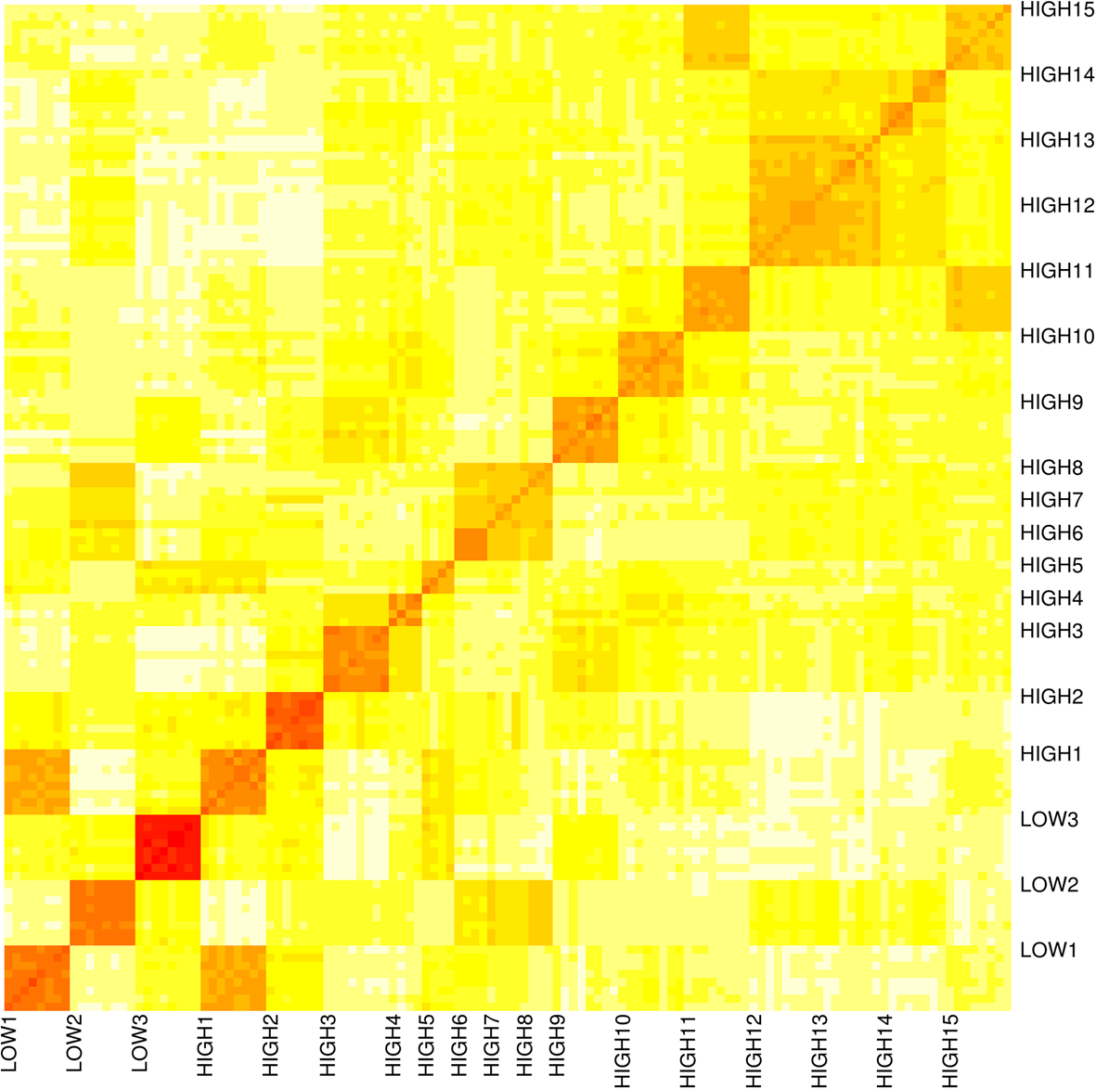
**Heatmap of genomic relationships between sugar beet individual plants.** Plants have been groupd by line. Darker colors indicate stronger genomic relationships.

Figure 1 shows the heatmap of estimated genomic relationships between individual plants. Plants have been ordered by root vigor (low and high) and line. Darker colours indicate closer kinship between individuals. The average genomic relationships was 0.365 (standard deviation 0.248; coefficient of variation 67.9%). The closest lines appeared to be “LOW1” and “HIGH1” $\left(\bar{a_{\text{ij}}}=0.938\right)$; the lines farthest apart were “HIGH2” and “HIGH13” $\left(\bar{a_{\text{ij}}}=0.104\right)$.

### Heritability and classification

Model fit was evaluated comparing the full (model (2)) and the reduced (null model: the intercept only) models through a likelihood ratio test. Deviance dropped significantly (p-value ≈0), showing good fit of the model. The estimated genetic variance was 11.856, on the liability scale. From Eq. 3 heritability was then estimated as *h*^2 ^= 0.783, with a standard error of 0.086.

From cross-validation, 500 testing subsets were generated. In each of these, observations were classified according to the model fitted to the corresponding training subset, and the classification error calculated as in (4), then averaged over all subsets. The estimated cross-validation error rate from (5) was 0.000731 (0.073%). Only 9 out of 12326 test observations (500 replicates for an average test set size of 24.65) were wrongly classified. All 9 missclassified observations belonged to a single low-root vigor line (line “LOW1”).

**Figure 2:**
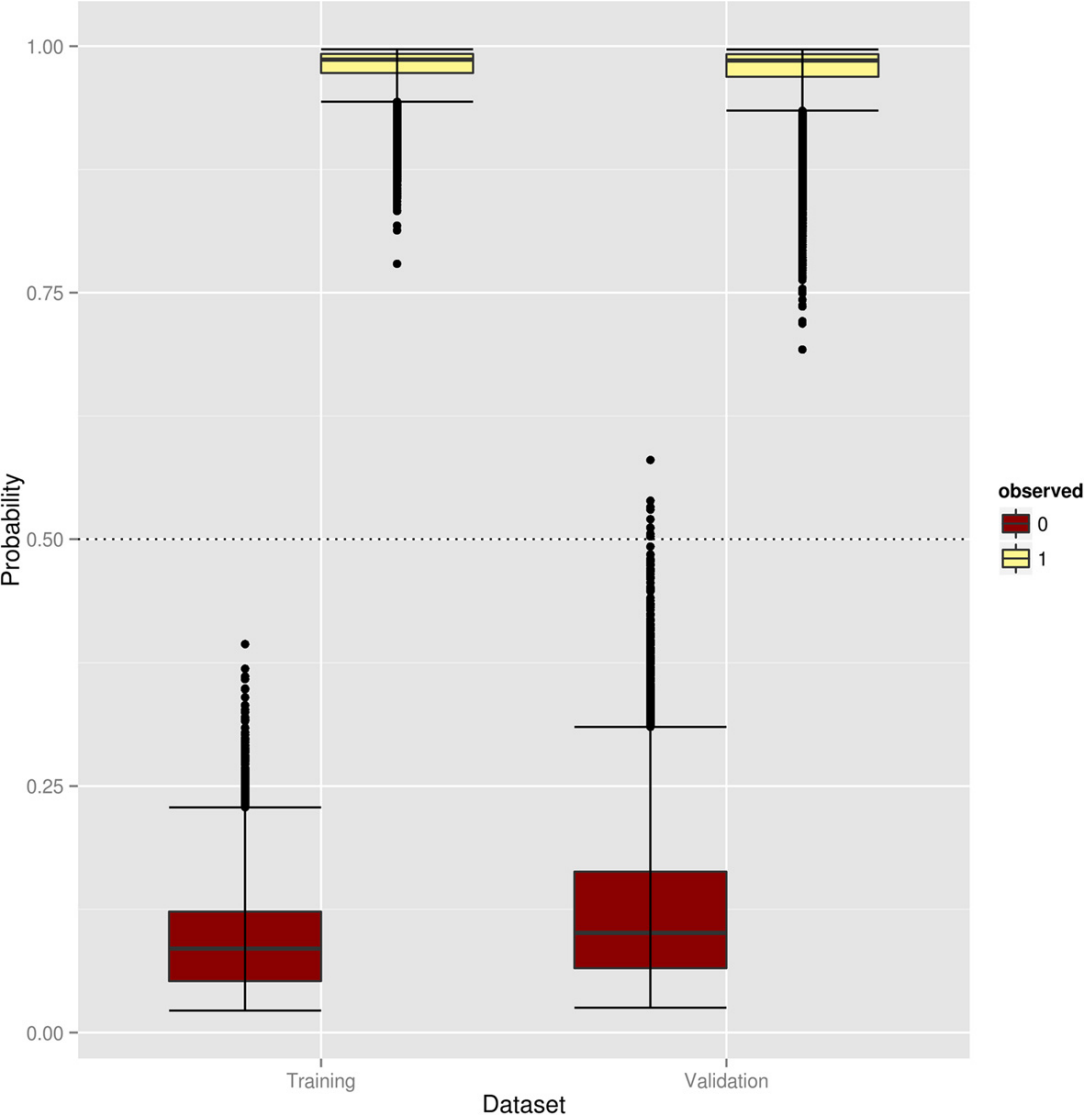
**Box plots of estimated probability vs observed root vigor in the training (left) and validation/test (right) data.** True high- and low-root-vigor individuals are in light yellow and dark red respectively. The dotted line is the classification threshold (*P *(*Y *= [ 0/1]|*X*) = 0.5).

In the training sets observations were always correctly classified (training error rate = 0). The average estimated probability of having low or high root vigor was calculated for each plant across all 500 replicates of the training and testing sets. The box plots in Figure 2 show the wider probability distribution in the test compared to the training data; the 9 misclassified observations in the test data are represented by points beyond the *P *(*Y *= 0|*X *)= 0.5 dotted threshold line.

## Discussion

In this paper, the problem of classifying binomial phenotypes using SNP markers in sugar beet (*B. vulgaris*) has been addressed. A very low cross-validation test error rate (<1%) was estimated for the genome-based classification of root vigor in sugar beet lines. Genomic predictions with different accuracies have been reported in literature: high (e.g. 0.89 for soluble solids content in apple trees [23]; 0.92 for fat and protein percentage in cattle [24]), moderate (e.g. ≈ 0.60 for egg weight in laying hens [25]) and low (e.g. 0.38 for stem height in loblolly pines [26]) accuracy of prediction. Wang et al. ([10]) reported accuracies ranging from 0.17 to 0.69 for a simulated categorical trait. In sugar beet, moderate to high prediction accuracies were estimated for a number of traits such as sugar content, molasses loss, root yield and mineral (*Na, K*) content [13,14].

The few prediction errors were all observed in line “LOW1”. This line had the strongest off-diagonal genomic relationship with line “HIGH1”. “LOW1” and “HIGH1” differ for root vigor (and related genes) but share most of their genetic basis. The close relationship of “LOW1” to “HIGH1” (a high root vigor line) may well explain why all 9 misclassifications were observed in this line, considering that SNP genotypes -through the genomic relationship matrix- were used as predictors. The error rate in line “LOW1” was nonetheless very low (∼1.1*%*).

The accuracy of genomic predictions is known to depend on a number of factors related to the nature of the analysed trait (e.g. heritability [3]) and to the experimental population at hand (e.g. sample size, number of markers, relatedness between the training and validation sets [3,27]).

Some relevant aspects are discussed below.

### Genetic architecture of the trait

The high predictive ability for root vigor estimated in this study (>99% of correct classifications) may be related to the heritability of the trait and to the number of segregating QTL underlying its expression: this is sometimes referred to as the “genetic architecture” of the trait [28].

**Figure 3:**
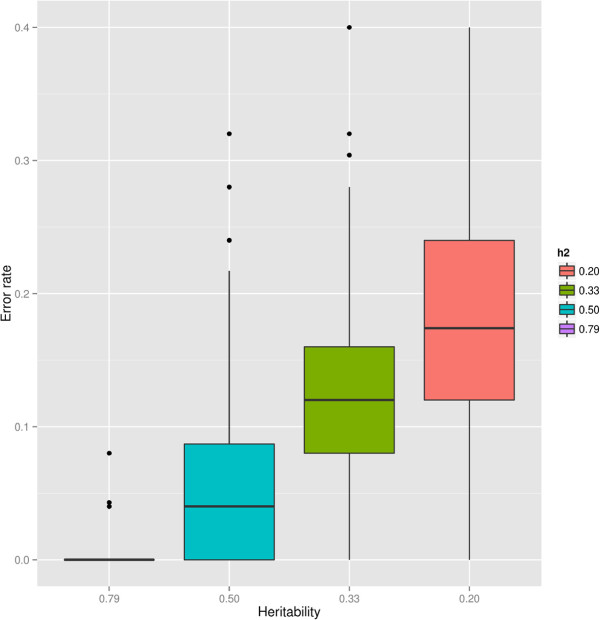
**Boxplot of the cross-validation test error rate for the original data (*h***^**2 **^**= 0 *. *** **783) and for lower heritabilities.**

Root vigor has been estimated to have high heritability (≈80*%*). For a highly heritable trait, using genetic markers to correctly predicting phenotypes is expected to yield good results. The effect of heritability on error rate was checked by fixing the heritability in model (2) instead of estimating it from the data. This has the effect of altering the covariance structure in the GBLUP mixed model equations from model (2) by loosening the relationship between genotypes and phenotypes. Artificially lower heritabilities of 0.5, 0.33 and 0.20 were tested by rerunning the analysis with the same cross-validation scheme and number of replicates. Figure 3 shows the boxplots of the error rate with the true heritability (utmost left column) and with progressively lower heritabilities: both the median error rate and the variance around it incresed when reducing the heritability. The error rate for *h*^2 ^= 0.5, 0.33 and 0.2 was 0.058 (5.8%), 0.128 (12.8%) and 0.181 (18.1%), respectively.

**Figure 4:**
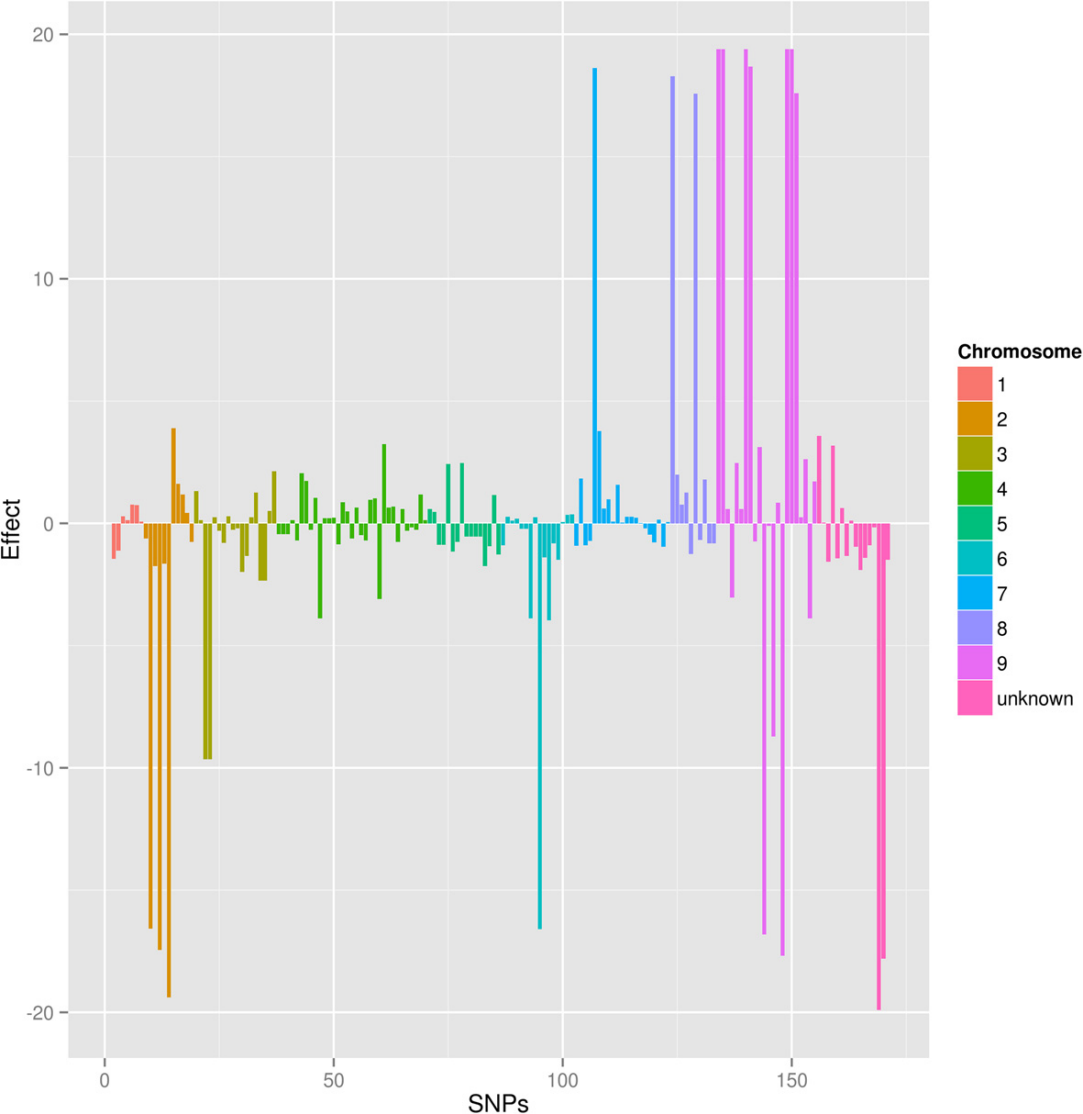
**Snp effects for root vigor along the genome of *B. vulgaris *.**

Not only the total genetic variance of a trait plays a role in determining the accuracy of genomic predictions, but also how this variance is spread along the genome (i.e. the distribution of QTL/genes underlying the trait). For a polygenic trait (i.e. determined by a large number of genes) a large number of markers would be needed to ensure that the genetic variance is fully captured. Contrariwise, for an oligogenic trait (i.e. determined only by few genes), even a small number of markers -as in the present study- may be sufficient to capture the genetic variance of the trait, provided that these markers are close to the relevant QTLs. A single-SNP genome-wide association study was performed in order to estimate marker effects for root vigor. A logistic regression model of the form *logit *(*p*_*i*_) = *μ *+ *SNP*_*m*_ (*p*_*i *_= *P *(*Y *= 1|*μ *+ *SNP*_*m*_); *SNP*_*m*_: individual genotype at SNP *m*) was fit to the data. The magnitude of estimated SNP effects is reported in the barplot in Figure 4. Large marker effects appear to cluster on specific scaffolds from a few chromosomes of the sugar beet genome, while most SNPs do not seem to have an appreciable effect on root vigor. Such distribution of marker effects agrees with an oligogenic basis for the trait root vigor. This, together with the high heritability of the trait, may help explain the very low classification error rate estimated with relatively few markers in this study.

### Linkage disequilibrium

The extent of linkage disequilibrium (LD) in the experimental population is a parameter relevant to the success of genomic predictions. The basic assumption underlying genome-wide predictions is indeed that observed genetic markers and unobserved QTLs are in LD [6]. LD between adjacent markers of around 0.2 -measured as *r*^2^[29]- is deemed to be required for reliable genomic predictions [3]. Dense marker panels ensure that there is sufficient LD between markers. With sparser panels this may not be the case. The available release of the *B. vulgaris* genome was not assembled in chromosomes, but organised in 82305 scaffolds (and contigs). The sugar beet genome sequence comprises 567 Mbps of which 85% could be assigned to chromosomes [30]. Most scaffolds -but not all- could therefore be mapped to chromosomes; however, the relative position of the scaffolds along the chromosomes was not known. Therefore, pairwise LD between adjacent SNPs could be estimated only within scaffold.

**Figure 5:**
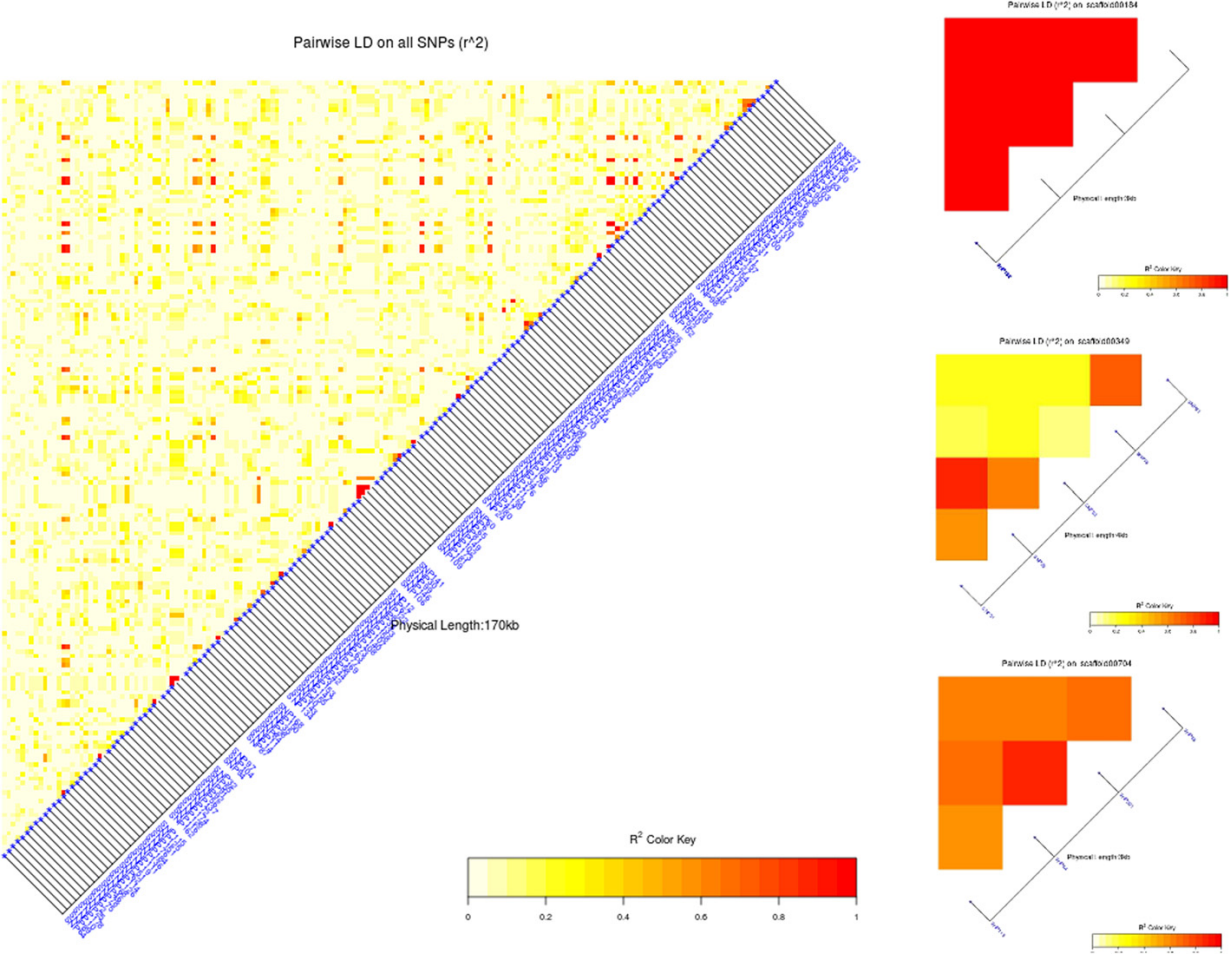
Linkage disequilibrium between all SNPs (left) and between SNPs on scaffolds 00184, 00349 and 00704 (right, from top to bottom).

The total average estimated LD between all pairs of markers, measured as *r*^2^, was 0.061. This values is below what is needed for genomic predictions to work, but refers to all markers, not only adjacent markers. Adjacent markers could be determined only within-scaffold; the average within-scaffold pairwise LD was *r*^2 ^= 0.404, which seems to be largely sufficient for reliable genomic predictions. Also the LD between markers with large effect on root vigor (see Figure 4) was estimated: this was *r*^2 ^= 0.327 on average, and can be interpreted as an indirect estimate of the LD between markers and QTLs.Figure 5 reports the LD heatmap between all markers (large panel on the left) and between markers on three scaffolds (small panels on the right): though no clear LD patterns emerge from the total set of SNPs, a strong LD structure is present on individual scaffolds.

### Imputation accuracy

Imputing missing genotypes is usually a preliminary step to the analysis of genomic data. After markers and individuals with low call-rate are edited out, there is usually still a small proportion of uncalled genotypes (e.g. < 5*%*) randomly distributed along the genome. Such missing genotypes are imputed using pedigree-based or pedigree-free methods. Imputation accuracy is typically very high; for instance, >95*%* correctly imputed genotypes were reported in maize [31] and cattle [32]. This usually applies to scenarios in which moderate to high density marker panels are available. With fewer markers genotype imputation may be less accurate, as a consequence of lower LD. This may be especially true for pedigree-free imputation methods, which rely heavily on between marker LD.

In order to estimate the accuracy with which genotypes were imputed in the present study, a subset with no missing genotypes was extracted from the total dataset. Increasing proportions of missing data were then artificially introduced in the data: 1%, 2%, 3%, 5%, 10% and 20%. For each proportion of missing genotypes, 5 random replicates were generated. The average proportion of correctly imputed genotypes over 5 replicates for each proportion of missing data was then used to estimate an empirical curve of the imputation accuracy. Results are summarised in Table 3: the intersection between the empirical curve and the percentage of missing genotypes in the original dataset (3.14%), provided an indirect estimate of the imputation accuracy obtained in this study: 0.840. This estimate appears to be quite robust, considering that up to 10% missing genotypes the empirical imputation accuracy curve is substantially flat, and only for missing data > 10*%* the accuracy of imputation seems to drop. An imputation error of about 16% is higher than what is typically found in humans and commercial crop and livestock populations. This may be due to the lower extent of LD estimated in this population with the availabe SNP panel, and to the lack of a mature assembly of the genome (partial information on chromosome structure and marker postion).

**Table 3:** Imputation accuracy with increasing proportions of missing genotypes

**% missing**	**1%**	**2%**	**3%**	**5%**	**10%**	**20%**
$\hat{\text{accuracy}}$	0.8405	0.8402	0.8402	0.8396	0.8301	0.8089

The average proportion (over 5 replicates) of correctly imputed genotypes ($\hat{\text{accuracy}}$) was used to estimate imputation accuracy.

### Comparison with another classification method

The threshold model used in this study for genome-enabled prediction of the binary trait root vigor in sugar beet was compared with Support Vector Machine (SVM), another widely adopted method for classification of categorical observations [20,33].

The kernel function and tuning parameter *C* to be used in SVM were chosen so to minimize the classification error through 5-fold cross-validation. A linear kernel ($K(x_{i},x_{i^{\prime }})=\langle x_{i},x_{i^{\prime }}\rangle =\overset{p}{\underset{j=1}{\sum }}x_{\text{ij}}x_{i^{\prime }j}$, for individual plant *i* and *p* parameters) and *C*=0.01 were chosen and used to classify sugar beet individual plants with SVM in the same cross-validation procedure adopted for the threshold model (5-fold, 100 repetitions). The estimated error rate was close to zero (0.025%), in line with what was obtained with the threshold model (0.073%). The two classifiers were compared also by looking at the ROC curves [34]: the two curves overlapped almost completely, having both an area under the curve (AUC) close to 1 (∼0.98). This shows that with both classifiers the total error rate and the number of false positives and false negatives were very low.

### Applications to sugar beet breeding

Root vigor, expressed as high root elongation rate, is essential for the efficient acquisition of mobile soil nutrients [35]; this is especially true in presence of water-nutritional stress [36]. The increased root elongation rate in response to low water availability or nutrient deprivation allow plants to circumvent water or nutrients limitations [37]. Of all sugar beet morphological root traits, root elongation rate shows the largest variation between high- and low-yielding genotypes and was shown to be significantly correlated with sugar beet yield [15].

Root traits are difficult to be measured accurately and this is an obstacle to reliable and effective selection. Genomic data can be used for early and accurate prediction of root vigor in sugar beet seeds, thereby enhancing the efficiency of breeding for rhizospheric stress tolerance and yield in sugar beet. Improvements are likely to come from shortened breeding cycles and more accurate and less expensive phenotypic evaluation.

## Conclusions

In this paper, the use of genomic information to predict a binomially distributed phenotype (root vigor) in sugar beet populations was presented. Prediction accuracy proved to be quite high, with an estimated cross-validation error rate close to zero (0.073%). Such excellent prediction performance may be related to properties of the analysed trait and available population. Root vigor was estimated to have high heritability (0.783) and to be determined by few genes with large effect. Despite the sparse SNP panel, there was sufficient within-scaffold LD where SNPs with large effect on root vigor were located. For an oligogenic highly heritable trait with a favorable distribution of markers on the genome, even with relatively few SNPs very accurate predictions can be achieved. The results described in this paper constitute an interesting application of genomic predictions to binomial (and more generally categorical/multinomial) traits, and may lead to promising applications of genomic selection in sugar beet breeding programmes.

## Competing interests

The authors declare that they have no competing interests.

## Authors’ contributions

FB carried out all statistical analyses and drafted most of the manuscript. PS, CB and MS selected the experimental population and generated all molecular and phenotypic data. AS contributed ideas to the work and drafted parts of the manuscript. All authors read and approved the final manuscript.
